# Personality of marathon runners: a narrative review of recent findings

**DOI:** 10.17179/excli2024-6907

**Published:** 2024-03-27

**Authors:** Lorin Braschler, Mabliny Thuany, Claudio Andre Barbosa de Lira, Volker Scheer, Pantelis T. Nikolaidis, Katja Weiss, Beat Knechtle

**Affiliations:** 1 Faculty of Medicine, University of Bern, Bern, Switzerland; 2 Center of Research, Education, Innovation and Intervention in Sport (CIFI2D), Faculty of Sport, University of Porto, Porto, Portugal; 3 Human and Exercise Physiology Division, Faculty of Physical Education and Dance, Federal University of Goiás, Brazil; 4 Ultra Sports Science Foundation, Pierre-Bénite, France; 5 School of Health and Caring Sciences, University of West Attica, Athens, Greece; 6 Institute of Primary Care, University of Zurich, Zurich, Switzerland; 7 Medbase St. Gallen Am Vadianplatz, St. Gallen, Switzerland

**Keywords:** marathon, ultra-marathon, psychology, personality, motivation, mental health

## Abstract

Participation in marathons has dramatically increased over the last few years. Marathon running has many proven beneficial effects, especially on cardiovascular health and fitness. Most research has focused on physiologic and pathophysiologic adaptations in connection with endurance exercise. Nevertheless, marathon running also has a major impact on psychological aspects and positively influences mental health, which has only recently attracted research interest. The present narrative review aimed to review the personality traits of marathon runners with an emphasis on recent literature. Marathon runners show a distinct personality and highly characteristic personality traits needed to successfully finish such a demanding race, i.e., a strong sense of vigor, self-sufficiency, and intelligence as well as low scores in anger, fatigue, tension, and depression. Furthermore, personality differences are detectable between runners of different sexes, ages, and performance level groups. This has significant clinical implications for athletes, coaches and competition organizers, as these groups show different patterns of personality traits. Future studies should focus on changes in cognition and mood states pre-, during, and post-endurance events, as well as during training periods. Large-scale studies comparing personality differences by sex, age, and performance are also important for better clinical guidance.

See also the graphical abstract(Fig. 1).

## Introduction

Participating in a marathon race is a physical activity of increasing popularity resulting from the running boom during the decades following the 1970s (Scheerder et al., 2015[75]). It is estimated that around two million people took part in a marathon race in 2015 and thousands of races are organized annually with great popularity (Kaleta-Duss et al., 2020[29]). Accordingly, the scientific production in topics related to marathon runners has grown during this period. So far, the focus of scholarly papers has been on physiological aspects such as adaptations of the cardiovascular (O'Riordan et al., 2023[63]) and musculoskeletal system (Shu et al., 2022[76]), sleep (Nikolaidis et al., 2023[61]), and skin to running (Kliniec et al., 2023[34]), as well as performance characteristics such as aerobic fitness (Alvero-Cruz et al., 2020[1]; Denadai and Greco, 2022[9]), training (Haugen et al., 2022[23]) and pacing of runners (Casado et al., 2021[7]; García-Manso et al., 2021[15]). Furthermore, pathophysiological aspects of marathon running, such as musculoskeletal injuries (Vasiliadis et al., 2021[84]; Weinrich et al., 2022[88]), acute kidney injury (McCullough et al., 2011[52]), electrolyte imbalances (Klingert et al., 2022[33]), gastrointestinal disturbance (Pugh et al., 2018[68]), and medical emergencies (Breslow et al., 2021[6]; Finke et al., 2023[13]) have also been studied. 

On the other hand, psychological aspects such as personality have received less attention. Usually, personality is defined as the totality of characteristics that make a person unique (Nikolaidis et al., 2018[60]). In fact, it is conceivable that personality is associated with both participation and performance in marathon runners (Nikolaidis et al., 2018[60]). Previous studies have already shown that marathon runners differ significantly from the general population and other sports disciplines in terms of personality traits such as self-sufficiency (Hartung and Farge, 1977[22]), self-discipline (Nudel et al., 1989[62]), introversion (Hagberg et al., 1979[20]), and conscientiousness (Piepiora et al., 2019[65]). Also, mental health in endurance sports has increasingly been investigated (Roebuck et al., 2018[69]; Thuany et al., 2023[81]). Although the personality of marathon runners has already been studied concerning sex, age, and performance level (Nikolaidis et al., 2018[60]), no review study has been conducted on the topic during the last five years. Considering that participation in marathon races has increased substantially in recent years, it is important to evaluate the personality traits of marathon runners. Therefore, the present study aimed to comprehensively review the personality traits of marathon runners with an emphasis on recent literature and updated findings and to compare personality traits between sexes and with other sports disciplines.

## Materials and Methods

This study was conducted as a narrative review. According to a predefined search strategy, the relevant literature was searched (Baethge et al., 2019[3]; Moher et al., 2015[55]). Two of the most common databases in the field of health and sports sciences (i.e., MEDLINE Ovid (PubMed), and Scopus) were searched, for appropriate literature (Falagas et al., 2008[12]). Medical subject headings were combined with free-text words to generate the most precise search possible. Personality traits of ultra-marathoners and triathlon participants were additionally included in the search due to a lack of articles covering solely the personality of marathon runners and considering that marathon runners may also compete in ultra-marathon and triathlon races. We considered every race longer than the traditional marathon distance of 42.195 km as an ultra-marathon (Knechtle, 2012[35]). The following keywords were used in the literature search: “marathon*”, “marathon run*”, “ultra-marathon*”, “ultra marathon*”, “ultra-marathon run*”, “ultra marathon run*”, “triathlon*” and “personality*”. We included all studies discussing personality traits and psychological aspects in half-marathon, marathon, ultra-marathon, and triathletes. All studies published until October 2023 were considered. In total, we found 194 articles across the two databases. After initial screening (title and abstract) of the literature, 128 were excluded and 13 of which were duplicates. A total of 66 articles were selected for this review. We analyzed the literature according to the psychological profile, the role of sex, age, and performance level in order to provide a differentiated breakdown of possible influencing variables on personality traits.

## Psychological Profile

Marathon runners show significant differences in psychological state when compared to the general population (Nikolaidis et al., 2018[60]). Table 1(Tab. 1) (References in Table 1: Freund et al., 2013[14]; Goddard et al., 2019[18]; Hartung and Farge, 1977[22]; Hoffman and Krouse, 2018[25]; Jerome and Valliant, 1983[27]; Johnson et al., 2012[28]; Piepiora et al., 2019[65]; Roebuck et al., 2020[70]; Roeh et al., 2020[71]; Valliant et al., 1981[82]; Waśkiewicz et al., 2018[86]) summarizes the key findings and study characteristics of analyzed studies investigating the psychological profile of endurance athletes. 

In general, marathon runners exhibit a strong sense of vigor and are characterized by low scores in anger, fatigue, tension, depression, and confusion (Nikolaidis et al., 2018[60]). Compared to the general population, middle-aged marathon runners and joggers were significantly more imaginative, intelligent, reserved, self-sufficient, sober, shy, forthright, well-educated and had a high socio-economic status (Hartung and Farge, 1977[22]; Hoffman and Krouse, 2018[25]). The high levels of self-sufficiency and imagination are most likely a result of running as there is a proven association between self-sufficiency and improved fitness (Hartung and Farge, 1977[22]). Interestingly, there are several studies describing marathon runners as rather reserved and introverted to some extent (Hagberg et al., 1979[20]; Hartung and Farge, 1977[22]; Jerome and Valliant, 1983[27]; Roeh et al., 2020[71]). Especially compared to other athletes, marathon runners exhibit higher introversion scores (Hartung & Farge, 1977[22]). However, in other studies, endurance athletes were shown to be more extroverted or no differences between the general population could be derived at all (Jerome & Valliant, 1983[27]; Roeh et al., 2020[71]). Ultra-marathoners were shown to have lower scores in affiliative extraversion which covers social warmth, affectionateness, and the tendency to value close relationships (Roebuck et al., 2020[70]). Furthermore, experienced ultra-marathoners were more skeptical about the goodness of human nature (McCutcheon and Yoakum, 1983[53]). Long-distance runners were characterized by an average level of neuroticism, extraversion, openness to experience and a high level of conscientiousness (Piepiora et al., 2019[65]). In another study, athletes were described as more self-motivated, extroverted, experience-seeking and less disinhibited (Roeh et al., 2020[71]). 

Marathon runners also exhibited personality differences compared to other endurance athletes. Higher scores in affiliation, life meaning and lower scores in the areas of weight concern, personal goal achievement and self-esteem were found in ultra-marathon participants compared to runners of shorter distances (Waśkiewicz et al., 2018[86]). Long-distance runners compared to joggers were more intelligent, self-sufficient, and tenderminded and reported less depression, confusion and more vigor (Jerome and Valliant, 1983[27]; Nikolaidis et al., 2018[60]). Marathon runners differed from joggers in being more reserved, intelligent, serious, tough-minded, practical, forthright, self-assured, tender-minded, imaginative, and self-sufficient, whereas joggers were more happy-go-lucky, apprehensive, controlled, conscientious, and less assertive (Nikolaidis et al., 2018[60]; Valliant et al., 1981[82]). Marathon runners compared to cross-country skiers were found to be significantly older, more intelligent, tender-minded, subjective, and creative (see Table 1(Tab. 1)) (Jerome and Valliant, 1983[27]). Compared to marathon runners, cyclists showed a similar psychological profile as cyclists exhibited significantly higher introversion and vigor scores and were lower on the tension, depression, anger and confusion scales compared to the general population (Hagberg et al., 1979[20]). Marathon runners scored slightly lower on extraversion and neuroticism than football players but higher in conscientiousness (see Table 1(Tab. 1)) (Piepiora et al., 2019[65]). 

After all, these changes in psychological traits indicate favorable changes in terms of mental health (Hagberg et al., 1979[20]). Endurance sports have been a proven important factor in the prevention of depressive symptoms and the improvement of cognitive symptoms (Roeh et al., 2020[71]). In this context, running was shown to provide several beneficial emotional impacts such as relief of tension, improved mood and self-image as well as creative episodes (Hoffman and Krouse, 2018[25]). These observations are supported by findings of endurance athletes, which showed significantly lower levels of anxiety, vulnerability, and depression than non-athletes (Goddard et al., 2019[18]; Hartung and Farge, 1977[22]; Roeh et al., 2020[71]). Marathon runners suffered fewer physical and cognitive complaints such as headaches, cognitive complaints and thought dysfunction than non-active controls (see Table 1(Tab. 1)) (Roeh et al., 2020[71]). 

Most ultra-marathon participants gain a clear benefit for themselves far beyond the physical (Hoffman & Krouse, 2018[25]). Ultra-marathoners who would not stop running even if it were bad for their health tend to be younger, less likely to be married, to have fewer children, a lower health orientation but higher personal goal achievement, psychological coping and life meaning (see Table 1(Tab. 1)) (Hoffman and Krouse, 2018[25]). Relatively high scores for health orientation, personal goal achievement, psychological coping, life meaning, and self-esteem are found in ultra-marathoners (Hoffman and Krouse, 2018[25]). Towards endurance exercise, these participants are highly task-oriented and present relatively high scores for health orientation, personal goal achievement, psychological coping, life meaning and self-esteem (Hoffman and Krouse, 2018[25]). 

Endurance exercise seems to ameliorate cognitive function probably as a result of enhanced neuronal plasticity or improved central vascularization (Roeh et al., 2020[71]). Marathon runners seem to have a less distrustful personality than the general population, indicated by a lower result of aberrant experiences (Roeh et al., 2020[71]). Additionally, lower scores of demoralization, helplessness, hopelessness, stress and feelings of worry have been identified in endurance athletes (Roeh et al., 2020[71]). These findings might be connected to mental toughness, a personality trait commonly found in athletes such as marathon runners (Goddard et al., 2019[18]). Mental toughness is considered one of the most important psychological components of athletic success, as it helps athletes boost self-confidence (Goddard et al., 2019[18]). However, mental toughness in combination with vigorous physical activity was found to be associated with the so-called “dark triad”, a personality complex consisting of Machiavellianism, narcissism, and psychopathy (Goddard et al., 2019[18]). Nevertheless, these attributes were not significantly found to be different in endurance athletes compared to the general population (Goddard et al., 2019[18]). Ultra-marathon runners are characterized by higher levels of extraversion, mental toughness, openness to experience and intellectual curiosity (see Table 1(Tab. 1)) (Goddard et al., 2019[18]). Athletes seem psychologically more resilient than sedentary controls (Roebuck et al., 2020[70]). Partially, this might be explained by the fact that ultra-marathon runners dispose of emotional regulation strategies such as cognitive reappraisal (Roebuck et al., 2020[70]). Additionally, compared to non-runners, ultra-marathon athletes react less physiologically to negative stimuli. However, it is not entirely clear whether endurance sports cause this fact or rather predispose individuals to participate in this kind of physical activity (Roebuck et al., 2020[70]). Both marathon and ultra-marathon runners were shown to have higher levels of self-efficacy compared to controls (see Table 1(Tab. 1)), which is a personality trait associated with highly challenging task performance, high ambitions and a strong commitment to achieving them (Freund et al., 2013[14]; Johnson et al., 2012[28]). 

A further psychological trait commonly found in marathon runners is a hardy personality that summarizes a high understanding and appraisal of commitment, control and challenges (Sánchez, 2009[72]). This significantly impacts both motivation and performance and enables athletes to reach goals and perform to the highest standards (González-García and Pelegrín, 2020[19]; Sánchez et al., 2009[73]). In both adolescent and adult marathon runners, higher levels of hardy personalities were detected compared to the age-matched sedentary control groups (Sánchez et al., 2009[73]). Although parental education styles were shown to influence personality traits in children, no association was found with hardy personality in this regard (González-García and Pelegrín, 2020[19]). 

In conclusion, the combination of low anxiety, mental toughness, self-efficacy, hardy personality, and efficient emotion regulation strategies enables marathon runners to cope with the immense psychological stress of a marathon or ultra-marathon race and still reach high performance. 

## The Role of Sex

Considering that more and more women are participating in long-distance events such as marathons and ultra-marathons (Knechtle, 2012[35]), we also reviewed personality variables between sexes. Most important findings alongside the study characteristics of the analyzed studies investigating differences in endurance athletes regarding sex are depicted in Table 2(Tab. 2) (References in Table 2: Gauld et al., 2023[17]; Karr et al., 2013[30]; Krouse et al., 2011[39]; Lantz et al., 2004[40]; López-Fernández et al., 2014[43]; Malchrowicz-Mośko and Poczta, 2018[48]; Nikolaidis et al., 2019[58]; Owens and Slade, 1987[64]; Pierce et al., 1997[66]; Poczta et al., 2021[67]; Weight and Noakes, 1987[87]).

Indeed, about 20 % of ultra-marathon finishers are women (Knechtle, 2012[35]), whereas almost 30 % of triathlon competitors are women (Poczta et al., 2021[67]). Female ultra-marathoners trained an average of 12.5 hours per week and spent 64 % of their training time alone (Krouse et al., 2011[39]). Approximately 40 % of female ultra-runners had children and 75.7 % reported working full-time, averaging 41.0 hours per week (Krouse et al., 2011[39]). Taken together, all of these characteristics can cause psychological demands on women that deserve to be investigated.

Over the past years, diverse studies have linked eating disorders and menstrual irregularities to female endurance runners (Nikolaidis et al., 2018[60]). In female ultra-marathoners, menstrual irregularities have been experienced by almost 40 % of runners during intensive training phases or due to emotional stress of competitions (Van Gend and Noakes, 1987[83]). However, these were only transient changes, as menstrual irregularities normalized once these stresses were cut out (Van Gend and Noakes, 1987[83]). Underdeveloped and distorted body image could be found in both male and female adolescent marathon runners, as well as concomitant anorexia nervosa and even (see Table 3(Tab. 3); References in Table 3: Gauffin et al., 2019[16]; Hartung and Farge, 1977[22]; Malchrowicz-Mośko et al., 2020[45][46]; Nikolaidis et al., 2019[58]; Nudel et al., 1989[62]) (Nudel et al., 1989[62]). It is conceivable that long-distance running, especially competitions exacerbates personality traits such as oversensitivity, tension, and compulsive self-discipline in individuals at risk (Nudel et al., 1989[62]). A study found abnormal eating attitudes symptomatic of anorexia nervosa in 14 % of female long-distance runners (Weight and Noakes, 1987[87]). However, only 5 % of them had low body mass and a prior history of amenorrhea and exclusively 0.8 % had been treated for anorexia nervosa (see Table 2(Tab. 2)) (Weight and Noakes, 1987[87]). Another study investigating elite female ultra-marathon runners found features of anorexia nervosa in 20 % of runners (Roebuck et al., 2018[69]). A further study applying the Female Athlete Triad Screening Tool to female ultra-marathon runners identified 5.2 % of runners indicative of a clinical eating disorder. Meanwhile, 26.8 % showed scores suggestive of subclinical eating disorder (Roebuck et al., 2018[69]). Nevertheless, these numbers showed no increased incidence of abnormal eating attitudes or anorexia nervosa in female long-distance runners compared to the general population (Weight and Noakes, 1987[87]). Other studies found similarities in psychological traits between anorexia nervosa patients and marathon runners, especially in terms of perfectionism, introversion and asceticism (Siegel et al., 1990[77]). Anorectic patients showed higher levels of perfectionism and lower satisfaction levels than general controls (Owens and Slade, 1987[64]). Similarly, female marathon runners scored higher on perfectionism, however, these high standards were not associated with dissatisfaction (see Table 2(Tab. 2)) (Owens and Slade, 1987[64]). Rather, this suggests that female marathoners use these high ideals in terms of achieving high goals relating to health and competitive success in such a way that is psychologically healthy and do not share the negative body image and abnormal notion towards food characteristics compared to female patients suffering from eating disorders (Owens and Slade, 1987[64]). 

Recently, symptoms of eating disorders in marathon runners have also been associated with obligatory exercise and exercise identity (see Table 2(Tab. 2)) (Karr et al., 2013[30]; Lantz et al., 2004[40]; Roebuck et al., 2018[69]). Among ultra-marathoners, the prevalence of exercise dependence is estimated at approximately 3.2-17 % (Roebuck et al., 2018[69]). Interestingly, there seems to be a positive correlation between exercise addiction and the length of running events, as marathoners exhibit higher addiction scores than 5-km runners and ultra-marathon scores even higher than marathoners (Roebuck et al., 2018[69]). In ultra-marathon runners, exercise addiction was shown to increase the risk for injuries, illnesses, overtraining syndrome, burnout, eating disorders and more severe pain than people who are not dependent on physical activity (Gauld et al., 2023[17]). Marathon runners reported with obligatory exercise also showed higher scores on abnormal eating attitudes than non-obligatory-exercising counterparts (Karr et al., 2013[30]). Particularly, female marathon athletes with exercise obsession were associated with high body dissatisfaction, which might lead to eating disorders (Karr et al., 2013[30]). Younger individuals seem at risk for developing obligatory exercise cognition and behaviors as they are more likely to maintain rigorous physical training (Karr et al., 2013[30]). Furthermore, obligatory exercise has also been linked to exercise identity, a construct of the incorporation of exercise into one's identity role with the potential for aberrant or obsessive attitudes (Karr et al., 2013[30]). Runners with high exercise identity reported more behaviors of abnormal eating attitudes and higher injury tolerance than persons with low exercise identity (Lantz et al., 2004[40]). Compared to male marathon runners, female athletes showed significantly higher exercise dependence scores when matched by the training volume (Pierce et al., 1997[66]). Similar to previous results, female ultra-marathoners with higher exercise identity were more likely to report abnormal eating behaviors and higher training intensity levels (see Table 2(Tab. 2)) (Lantz et al., 2004[40]). Therefore, endurance runners might have increased risks of developing abnormal eating behaviors (Lantz et al., 2004[40]). Nevertheless, ultra-marathoners do not seem as absorbed with eating attitudes and body issues, as reported by other authors (Lantz et al., 2004[40]). 

Only little research has been done concerning metacognition i.e., how accurately marathon runners predict their performance (Liverakos et al., 2018[42]). The inability to set realistic goals will lead to suboptimal performance, such as starting a race too fast and negatively impacting confidence and motivation (Liverakos et al., 2018[42]). Typically, higher overconfidence is observed in men, while women are more likely to have a more realistic prediction of their performance (Liverakos et al., 2018[42]). Female runners were shown to pace more evenly during marathon races, whereas men tend to be overconfident by starting too fast (Amirkhanyan et al., 2021[2]). Uneven marathon pacing often leads to worse performances in competitions (Amirkhanyan et al., 2021[2]). Usually, women are more aware of their body composition compared to their male counterparts (Liverakos et al., 2018[42]). In endurance athletes, men were 2.4-times as likely to misjudge their body mass index (BMI) compared to women (Liverakos et al., 2018[42]). However, a recent study measuring the accuracy of self-reported anthropometric data by marathon runners found recreational marathoners to underreport their body mass, leading to underestimated BMI (Nikolaidis and Knechtle, 2020[59]). Female runners tended to underreport their body mass to a greater extent than male runners, whereas male marathoners overreported their height (Nikolaidis and Knechtle, 2020[59]). A recent study investigating the accuracy of race-time predictions in half-marathon participants and the actual performances found an overall tendency towards a good calibration (Liverakos et al., 2018[42]). Interestingly, women showed higher overconfidence and were more likely to predict faster race times than was achieved. Nevertheless, similar results between self-esteem and anxiety levels between male and female marathon runners were found (Estok and Rudy, 1987[11]). 

Physical exercise such as marathon running has several proven benefits for physical and psychological health (Nikolaidis et al., 2018[60]). Among others, physical activity before, during and after cancer diagnosis improves outcomes for breast cancer as it protects against recurrence and progression in breast cancer survivors (Malchrowicz-Mośko, 2022[44]). Regular physical exercise in breast cancer survivors improves the quality of life and immune system function and reduces cancer-related fatigue and nausea (Malchrowicz-Mośko, 2022[44]). Additionally, positive changes in personality functioning, locus of control, mood states such as anxiety and depression, perceived physical competence, self-esteem and satisfaction with life has been shown to be ameliorated by physical activity (Malchrowicz-Mośko, 2022[44]). In breast cancer survivors, running improves stress reduction and reduces the risk of cancer recurrence (Malchrowicz-Mośko, 2022[44]). Compared to healthy women, breast cancer survivors showed different motivations for running (Malchrowicz-Mośko, 2022[44]). Mean motivational factors for breast cancer survivors were weight concern, psychological coping, recognition, self-esteem and life meaning compared to healthy women whose principal motivations were health orientation, personal goal achievement and affiliation (Malchrowicz-Mośko, 2022[44]). 

Recently, several studies have examined backgrounds and motivations for participation in endurance events such as marathon running (Roebuck et al., 2018[69]). Compared to athletes of different sports, ultra-marathoners were more committed to running, confident, competitive and goal-oriented but less win-oriented (Roebuck et al., 2018[69]). Female ultra-runners scored significantly higher in task orientation than ego orientation compared to other ultra-marathoners (Roebuck et al., 2018[69]). In contrast to women, male marathon runners' primary motivations were to increase speed, reach their potential, personal goal achievement and competitive reasons (Yang et al., 2022[90]). In triathlon participants, women demonstrated significantly more often the will to feel unity and integration as well as the desire to gain recognition from others compared to men (Poczta et al., 2021[67]). In contrast, men valued the desire to feel equal significantly higher compared to female triathlon participants (Poczta et al., 2021[67]). Female triathlon participants also assigned a significantly higher value to ambitions to win a high rank in sports events than men (Poczta et al., 2021[67]). Additionally, the desire to escape everyday life and the prevailing fashion were more important for women than male triathlon participants (Poczta et al., 2021[67]). Male triathlon participants exhibited the highest scores in logical thinking compared with participants of different sports (Skurvydas et al., 2022[78]). Recent studies investigating motives for taking up running in men and women found the most important reasons for female runners to be weight concern, self-esteem, affiliation, psychological coping and life meaning (see Table 2(Tab. 2)) (Malchrowicz-Mośko et al., 2020[47]; Malchrowicz-Mośko and Poczta, 2018[48]). On the other hand, male runners were rather motivated by competitive reasons (Malchrowicz-Mośko et al., 2020[47]). Another study found that the highest motivations to run a marathon were intrinsic (meaning of life, self-esteem, health orientation), whereas extrinsic or ego-related motivations were the lowest (Nikolaidis et al., 2019[58]). Interestingly, female runners had higher scores in self-esteem, achieving personal goals, affiliation with other runners and weight concerns than male marathon runners (Nikolaidis et al., 2019[58]). Furthermore, female marathoners, compared to male runners, scored higher in coping, self-esteem and goal achievement (Nikolaidis et al., 2019[58]). Comparing female marathoners to female ultra-marathon participants, marathon runners showed higher health orientation and outscored ultra-marathoners for competitive reasons (Nikolaidis et al., 2019[58]). Men were disproportionally identified as obligatory runners (Malchrowicz-Mośko and Poczta, 2018[48]). Male obligatory runners were characterized by a high desire to achieve recognizable success, whereas male recreational runners more heavily upheld the motive of physical well-being (Malchrowicz-Mośko and Poczta, 2018[48]). Meanwhile, female runners reported greater benefits from running through opportunities to meet people, relief from depression, and feeling less shy (Malchrowicz-Mośko and Poczta, 2018[48]). In general, the desire to get away from everyday life, to maintain good physical condition and health, to test themselves, to develop a passion for running, and to achieve an affirmed goal was important equally important for both men and women (see Table 2(Tab. 2)) (Malchrowicz-Mośko and Poczta, 2018[48]). However, the desire to feel strong emotions associated with participation in sporting events and the desire to have good fun was more important for women than for male runners (Malchrowicz-Mośko and Poczta, 2018[44]). On the other hand, the desire to maintain good physical condition and health was more important for men than for women (Malchrowicz-Mośko and Poczta, 2018[44]). In female ultra-marathon runners, the main source of motivation was general health and personal achievement (Krouse et al., 2011[39]). Furthermore, compared to male ultra-runners, women were more task-oriented than ego-oriented, most set goals for their events and coaches were rarely used (Krouse et al., 2011[39]). In triathletes, no differences in general motivational motives between men and women were found (López-Fernández et al., 2014[43]). However, lower amotivation scores were reported in women compared to men (López-Fernández et al., 2014[43]). Nevertheless, amotivation scores were very low both for male and female triathlon participants (López-Fernández et al., 2014[43]). Low amotivation is associated with positive consequences in sports such as high commitment (López-Fernández et al., 2014[43]). 

To summarize, there are identifiable differences in the personality of endurance runners regarding sex. Eating disorders have been a major focus of research. However, no increased incidence of anorexia nervosa was found, suggesting high goal growth of female endurance athletes, which happens in a psychologically healthy manner. Nevertheless, studies comparing personality characteristics between women and men are relatively rare, warranting further studies in this field. 

## The Role of Age

Endurance sports such as marathon running are popular among a wide variety of age groups (Knechtle, 2012[35]; Nikolaidis et al., 2018[60]), despite differences in the frequency of participation. Considering that runners adopt different characteristics over life - associated but not determined by age - it would be of great clinical importance to identify possible differences between different age groups of marathon runners (Nikolaidis et al., 2018[60]). Table 3(Tab. 3) summarizes most relevant findings and study characteristics of analyzed studies investigating age differences in context of personality traits in endurance athletes.

Most ultra-marathoners are in their mid-30s to mid-50s, with a mean age of 45 years, most of the runners are men and the majority are married compared to the general population (Hoffman and Krouse, 2018[25]; Roebuck et al., 2018[69]). Furthermore, ultra-marathoners tend to be well-educated, work in white-collar professions and have a comparably low incidence of various self-reported medical and psychological disease (see Table 1(Tab. 1)) (Hoffman and Krouse, 2018[25]; Roebuck et al., 2018[69]). The mean age of successful finishers in ultra-marathon runners is approximately 45 years (Knechtle, 2012[35]). Nevertheless, the age of peak performance differs between marathon and ultra-marathon runners (Knechtle, 2012[35]). Elite marathon runners reach peak performance within 30 years, whereas male ultra-marathon athletes achieve best times between 30 and 49 years of age and female ultra-marathoners between 30 and 54 years (Knechtle, 2012[35]). Compared to joggers, marathon runners are older, run a higher distance per week and train for a longer period of time (Valliant et al., 1981[82]). Marathoners were also significantly older than cross-country skiers (Jerome and Valliant, 1983[27]). Interestingly, no significant age-related decline in marathon and half-marathon performance could be observed before age 55 in a study investigating middle and old-aged runners (Leyk et al., 2010[41]). After that, only a moderate decline could be detected (Leyk et al., 2010[41]). Indeed, 25 % of the 65- to 69-year-old runners were faster than 50 % of the 20- to 54-year-old runners (Leyk et al., 2010[41]). A recent study showed the mean score of expectations regarding aging amongst marathon runners to be relatively high compared to controls (Koronios et al., 2017[37]). Moreover, the exercise frequency per week was associated with the participants' age, suggesting a positive influence of exercise frequency and general health on expectations regarding ageing (Koronios et al., 2017[37]). 

There have been controversial opinions about long-distance running in preadolescents or early adolescents (see Table 3(Tab. 3)) (Nudel et al., 1989[62]). A study investigating athletes aged between 8- to 18 years found comparable personality traits to adult long-distance runners with high values for boldness, warmth, conformity, sensitivity, dominance, high drive with tension, self-discipline, emotional stability and intelligence (Nudel et al., 1989[62]). However, there were signs of distorted body image suggesting the development of negative personality traits and possibly psychological disorders warranting the need for psychological screening of young children participating in strenuous training programs (Nudel et al., 1989[62]). 

A study comparing marathon runners of a 40- to 49-year age group to a 50- to 59-year age group found a higher fitness level in both groups compared to non-marathoners (Hartung and Farge, 1977[22]). Regarding differences in personality, no significant differences between the two age groups could be identified (see Table 3(Tab. 3)) (Hartung and Farge, 1977[22]). However, the younger group tended to express higher scores in self-sufficiency (Hartung and Farge, 1977[21]). Nevertheless, compared to sedentary controls, middle-aged runners and joggers showed high levels of self-sufficiency and imagination and tended toward introversion in terms of personality traits (see Table 3(Tab. 3)) (Hartung and Farge, 1977[21]). 

Generally, endurance athletes show a tendency for sensation seeking and have a high need to achieve a high quality of life and the feeling of joy (Poczta et al., 2021[67]). This is particularly pronounced among younger individuals and those with a higher education level (Poczta et al., 2021[67]). The most important factor for ultra-marathon participants was a drive to explore their physical and mental limits (Roebuck et al., 2018[69]).

In terms of metacognition, older runners more accurately predicted their race time than their younger counterparts, indicating better calibration in older athletes (Liverakos et al., 2018[42]). Similarly, more experienced marathoners were more accurate in their race time prediction compared to first- or second-time marathon finishers (McKelvie et al., 1985[54]). Nevertheless, being younger than 50 years was significantly associated with reaching their desired performance outcome (Gauffin et al., 2019[16]). Furthermore, young runners were shown to be more pleased with their achieved result (Gauffin et al., 2019[16]). 

In recent years, there has been increased research into what drives marathon runners to pursue their sport (Nikolaidis et al., 2018[60]). Younger male marathon runners valued competitive reasons higher compared to older runners, as a recent study could show (see Table 3(Tab. 3)) (Nikolaidis et al., 2019[58]). Male marathon runners in the <30-year-age group compared to the 35- to 40-year and 40- to 45-year-age group scored higher in “competing with other runners” (Nikolaidis et al., 2019[58]). However, runners >50 years scored higher in general health orientation, body mass concern, life meaning and affiliation with other runners and lower in personal goal achievement compared to runners in the 20- to 28-year age group (Nikolaidis et al., 2019[58]). Another study found a decreased level of motivation due to personal goal achievement, competition and recognition scales with increasing age (Malchrowicz-Mośko et al., 2020[47]). Furthermore, younger runners were rather focused on results while older runners were more drawn to social interaction in mass running events such as a half-marathon (Malchrowicz-Mośko et al., 2020[46][47]). Younger runner's motives for taking part in endurance events were often personal goal achievement. In contrast, older runners were rather motivated by life meaning, health, body mass concerns, or affiliation with other runners (see Table 3(Tab. 3)) (Malchrowicz-Mośko et al., 2020[46]). Similar differences were also found among different ages in triathlon participants (Malchrowicz-Mośko et al., 2020[46]). In contrast, among children aged 12 years, fun, interest and enjoyment were the most important factors for participating in running (Malchrowicz-Mośko et al., 2020[45]). Other studies identified five primary motives for children's participation in sports: perception of competence, fun and enjoyment, parents, learning new skills and friends (Malchrowicz-Mośko et al., 2020[45]). Although teenagers aged 13-18 are very sensitive to the influence of other people regarding motivation for participation in competitive sports, the quality of friendship had only a weak influence on self-esteem and commitment in sports (Malchrowicz-Mośko et al., 2020[45]). 

Conclusively, there are variations between different age groups and anthropometric data, metacognition, and motives for participation in endurance sports. Nevertheless, there is little research concerning personality differences between the age groups of endurance athletes.

## The Role of Performance Level

Marathon running is becoming progressively popular among recreational runners compared to elite athletes (Nikolaidis et al., 2018[60]). Also, half-marathon encouraged physical activity regardless of age and gender in previously physically inactive individuals (Malchrowicz-Mośko et al., 2019[49]). There are increasingly more finishers of marathons due to the increased participation of recreational runners rather than elite athletes (Nikolaidis et al., 2018[60]). A similar trend has been observed in ultra-marathon races (Knechtle and Nikolaidis, 2018[36]). This asks whether differences in personality traits can be distinguishable regarding performance level. In Table 4(Tab. 4) (References in Table 4: Brace et al., 2020[4]; Brandstätter et al., 2013[5]; Harris et al., 1989[21]; Howe et al., 2019[26]; Kędra and Łaguna, 2022[32]; Krokosz et al., 2018[38]; Malchrowicz-Mośko and Waśkiewicz, 2020[50]; Nicolas et al., 2019[57], 2022[56]; Stoeber et al., 2009[79]; Stolarski et al., 2022[80]; Waleriańczyk and Stolarski, 2021[85]) key findings alongside study characteristics of analyzed studies regarding differences in performance levels of endurance athletes are listed.

In South African marathon runners, a higher preference for mornings i.e., morning types in 66.7 % of runners, was associated with better results for half-marathon and marathon performances (Henst et al., 2015[24]). Morning-oriented runners trained on more days, for more hours per week, and seemed more physically active, suggesting an influence of chronotypes on physical activity and vice versa (Henst et al., 2015[24]). Another study found no significant differences between Type A and B personalities in finishing times or subjective stress during a marathon race (Elmore and Evans, 1985[10]). Ultra-marathoners scored higher on extraversion and openness to experience scales than normal controls, suggesting a high thrive for sensation seeking and experience of exciting and intentionally controlled risk (see Table 1(Tab. 1) and Table 4(Tab. 4)) (Kazimierczak et al., 2019[31]; Krokosz et al., 2018[38]). Furthermore, ultra-runners often showed a strong drive to explore their physical and mental limits (Wickström et al., 2019[89]). Ultra-marathon runners have higher emotional stability scores than the general population (Gauld et al., 2023[17]). In turn, they tend to be calm and even-tempered even when confronted with a difficulty or an unexpected situation (Gauld et al., 2023[17]). Half-marathoners with high-trait emotional intelligence performed better and showed faster completion times compared to those with low-trait emotional intelligence (Howe et al., 2019[26]). Training Pace and fastest 10-km race time correlated significantly with final race times in marathon runners (McKelvie et al., 1985[54]). 

Compared to the general population, marathon runners show significantly higher levels of hardy personality, which considerably influences both performance and motivational aspects of marathon running (Sánchez, 2009[72]). Furthermore, it can differentiate between marathon participants with the best and worst records (Sánchez, 2009[72]). Ultra-marathon runners were shown to have significantly higher scores of mental toughness compared to hockey, soccer, and tennis players, and mixed martial artists (Brace et al., 2020[4]). Mental toughness was significantly associated with self-efficacy (see Table 4(Tab. 4)) (Brace et al., 2020[4]). It is postulated that self-efficacy might buffer pain due to increased endogenous opioid release, thus increasing mental toughness (Brace et al., 2020[4]). 

Inevitably, there is also a certain amount of pain experienced during a long-distance endurance event like an ultra-marathon (Freund et al., 2013[14]; Nikolaidis et al., 2018[60]). In this context, a reduction of pain sensitivity has been shown during or immediately after exercise (Johnson et al., 2012[28]). In marathon runners higher pain threshold, pain tolerance and self-efficacy was found compared to sedentary controls (see Table 1(Tab. 1)) (Johnson et al., 2012[28]). Interestingly, pain-specific self-efficacy accounted for 40% of the pain tolerance difference between runners and sedentary controls (Johnson et al., 2012[28]). Recently, self-efficacy itself was shown to be a predictor of performance (Johnson et al., 2012[28]). In the fastest runners of a 161 km ultra-marathon race, a moderate exercise-induced analgesic effect was detectable after the race, as reported by pressure pain sensitivity (Roebuck et al., 2018[69]). Currently, this phenomenon is thought to be due to a combination of increased endogenous opiate release and other central pain-modulating processes (Johnson et al., 2012[28]). Interestingly, ultra-marathon runners have a significantly increased pain tolerance compared to controls (see Table 1(Tab. 1)) (Freund et al., 2013[14]). Participants of these events were also shown to be more spiritually accepting, explorative, self-transcendent and less cooperative, harm-avoidant and harm-dependent than the control group, contributing to a great eagerness in controlling physical needs and complaints for a higher goal (Freund et al., 2013[14]). Furthermore, ultra-marathon runners exhibited lower scores of pain-related anxiety than non-running controls (Wickström et al., 2019[89]). After all, low pain perception may predispose persons to participate in long-distance running (Freund et al., 2013[14]).

Recently, anxiety, perfectionism, self-efficacy, and positive orientation were identified as essential personality traits in sports and exercise (Kędra and Łaguna, 2022[32]). Positive orientation was positively associated with training engagement (see Table 4(Tab. 4)), which predicted marathon performance (Kędra and Łaguna, 2022[32]). Athletes with higher positive orientation were associated with higher training frequency, persistence, and training engagement, enabling successful race progression and better race performance (Kędra and Łaguna, 2022[32]). Training engagement was shown to predict marathon race times (Kędra and Łaguna, 2022[32]). Perfectionism, indicated by a strong pursuit of flawlessness and setting remarkably high standards, was shown to be a significant predictor of race results in a 10-km run, a half-marathon, and a triathlon (Stoeber et al., 2009[79]; Waleriańczyk and Stolarski, 2021[85]). In terms of sports science, perfectionist strivings have been associated with positive attitudes, beliefs, and processes, such as the hope of success, competitive self-confidence, self-serving attributions of success and failure, and lower levels of anxiety and burnout (Stoeber et al., 2009[79]). Especially during the training and preparation phase for a competition, perfectionist strivings are a key factor affecting performance (see Table 4(Tab. 4)) (Waleriańczyk and Stolarski, 2021[85]). In this context, youth soccer players with high scores of perfectionist strivings were shown to invest more time in sport-specific activities compared to their less perfectionist counterparts (Waleriańczyk and Stolarski, 2021[85]). Perfectionism was significantly associated with conscientiousness and correlated with personal best, anticipated and actual performance, sport level, and level of preparation for the competition (Waleriańczyk and Stolarski, 2021[84]). Similarly, perfectionist strivings are significantly associated with higher sports engagement characterized by a strong bond between the athlete and the sport in terms of generalized and positive cognitions, attitudes, and affective states (Stolarski et al., 2022[80]). Sport engagement is associated with lower levels of burnout, better self-regulation, conscientiousness, emotional stability, more frequent flow experience, and higher training hours (Stolarski et al., 2022[80]). Compared to recreational runners, professional and semi-professional athletes showed significantly greater vigor, absorption, and general engagement (see Table 4(Tab. 4)) (Stolarski et al., 2022[80]). 

Mood alterations during or after endurance races have been reported (Nikolaidis et al., 2018[60]; Roebuck et al., 2018[69]). Especially the so-called “runner's high” i.e., an exuberant feeling of “well-being” after running, is well known (Harris et al., 1989[21]). Increased endorphin levels likely cause it as exercise at sufficient intensity was shown to produce endogenous opiates (Harris et al., 1989[21]; Johnson et al., 2012[28]). On race day, scores of anxiety and hostility were significantly higher compared to rest days, whereas depression and libido scores showed no significant changes (see Table 4(Tab. 4)) (Harris et al., 1989[21]). Before the race, runners experienced significantly higher levels of energetic and tense arousal, whereas no changes in hedonic tone could be distinguished (Krokosz et al., 2018[38]). Extraversion was positively correlated to energetic arousal (i.e., perceived energy and vigor) and negatively associated with tense arousal (i.e., less stress) for pre-race levels (see Table 4(Tab. 4)). In contrast, neuroticism correlated positively with tense arousal and negatively with hedonic tone on pre-race examinations (Krokosz et al., 2018[38]). During an ultra-marathon race, psychological relaxation and flow state increased during the first hour of running but decreased afterward (Roebuck et al., 2018[69]). An increase in fatigue, exhaustion, and stress states and a decrease in recovery states, vigor, tension, mental stress, and arousal levels in response to sexually provocative images were found in runners after finishing an ultra-marathon race (Howe et al., 2019[26]; Krokosz et al., 2018[38]; Nicolas et al., 2019[57], 2022[56]; Roebuck et al., 2018[69]). Total mood disturbance scores were reduced in ultra-marathoners, recreational runners, and non-exercisers after a session of aerobic exercise. However, changes in total mood disturbance scores were most pronounced in ultra-marathoners (Roebuck et al., 2018[69]). Usually, these mood changes were resolved within one week up to one month after the race (Nicolas et al., 2019[57]; Roebuck et al., 2018[69]). Generally speaking, the harder the situation is, the longer the needed recovery time (Nicolas et al., 2022[56]). However, emotional intelligence was shown to have an important protective role against stress during endurance running (Nicolas et al., 2022[56]). Athletes with higher scores in emotional intelligence showed higher levels of recovery before the race, were able to handle psychological stress better and recovered more easily after the race (see Table 4(Tab. 4)) (Nicolas et al., 2019[57], 2022[56]). Furthermore, emotional intelligence was linked with emotional experience, emotion regulation and performance (Nicolas et al., 2019[57]). Lower scores in emotional intelligence were linked with high levels of anger and confusion during a 282-km ultra-marathon. In contrast, participants with high scores in emotional intelligence showed lower levels of anger, confusion, depression, fatigue and tension and high levels of calmness and happiness (Howe et al., 2019[26]; Nicolas et al., 2019[57]). In the month following the race, participants with high scores of emotional intelligence depicted significantly higher levels of happiness and excitement compared to counterparts with low levels of emotional intelligence (Nicolas et al., 2019[57]). 

The increasing physical and mental demands over the course of the race are associated with a concomitant increase in serum and salivary cortisol secretion (Brandstätter et al., 2013[5]; Harris et al., 1989[21]; Howe et al., 2019[25]). Mood changes on race day were shown to be significantly associated with cortisol increase (Harris et al., 1989[21]). This increase was more pronounced in athletes reporting an action crisis within 2 weeks before the race (see Table 4(Tab. 4)) (Brandstätter et al., 2013[5]). Additionally, these athletes showed poorer running performance than those without reported action crises prior to the race (Brandstätter et al., 2013[5]). Additionally, ultra-marathoners with higher scores in emotional intelligence exhibited a higher cortisol response, suggesting that the runners can push themselves to their physical limits better and are better prepared to handle the emotions experienced during an ultra-marathon (Howe et al., 2019[26]). Vitamin D3 supplementation has a proven positive influence on mood and is able to reduce symptoms of depression (Krokosz et al., 2018[38]). Vitamin D3 supplementation before an ultra-marathon affected pre-race mood stated significantly in a positive way (see Table 4(Tab. 4)), as it correlated with higher energetic arousal and vigor, whereas no significant effect was observable for post-race mood alterations (Krokosz et al., 2018[38]). 

Recently, increasing attention has been paid to cognitive processes and function during and after endurance races (Roebuck et al., 2018[69]). Contradictory results were reported concerning cognitive function (Roebuck et al., 2018[69]). Some studies found cognitive function to be reduced after an ultra-marathon, other studies found an improvement between pre- and post-race cognitive function, whereas other studies did not report any significant changes in cognitive function after ultra-marathon running (Roebuck et al., 2018[69]). However, olfactory function was reduced after an ultra-marathon suggesting cognitive impairment, as olfactory function acts as a surrogate marker of cognitive function (Roebuck et al., 2018[69]). Interestingly, faster runners scored better and were more accurate on inhibitory control tasks requiring motor inhibition and prospective memory task than slower runners (Roebuck et al., 2018[69]). 

Approximately 50 % of ultra-marathon participants reported experiencing primarily internally focused thoughts (i.e., sensations arising from the lower limbs or associated with respiration), whereas 50 % experienced largely externally focused thoughts (i.e., dissociative cognitive processes) (Roebuck et al., 2018[69]). Several different cognitive strategies used by ultra-runners have been reported, such as visualization, reading, pre-race paraphernalia, setting goals, self-talk and thought control (Roebuck et al., 2018[69]). Another study found that only a small number of elite endurance athletes (6 %) used dissociative coping strategies compared to 69 % of recreational runners (McKelvie et al., 1985[54]). Elite marathon runners, therefore can closely monitor their bodies for signals of distress and adjust their pace in an appropriate way (McKelvie et al., 1985[54]). The use of associative coping strategies was significantly associated with higher pain tolerance, whereas dissociative strategies and catastrophizing reduced pain tolerance (Johnson et al., 2012[28]). Emotional intelligence was significantly associated with the use of more efficient coping strategies (i.e., task-oriented coping) in terms of stress management, less anxiety, perceived control, and performance satisfaction in ultra-marathon runners (Howe et al., 2019[26]; Nicolas et al., 2022[56]). Athletes with high trait emotional intelligence were more competent in coping and perceived less stress but more well-being and pleasant emotion states (Nicolas et al., 2022[56]). 

Overuse injuries in endurance runners are a common problem severely impairing training and race performance (Knechtle and Nikolaidis, 2018[36]; Wickström et al., 2019[89]). A recent study found that approximately 41 % of marathon runners sustained a prolonged injury during the training phase of a race (Gauffin et al., 2019[16]). Additionally, self-determined motivation for marathon running may act as a factor in preventive behaviors regarding injury (Chalabaev et al., 2017[8]). Severe illnesses (i.e., malignant hyperthermia, rhabdomyolysis and hyponatremia) that require hospitalization with intensive care after an ultra-event are rare, representing 0.2-0.4 % of ultra-endurance runners (Gauld et al., 2023[17]). Studies suggest that runners who display signs of exercise addiction may be at increased risk of needing intensive care after an ultra-endurance event (see Table 2(Tab. 2)) (Gauld et al., 2023[17]). A recent study investigating ultra-runners who needed intensive care after an ultra-endurance event found that only one out of the 12 ultra-runners presented an at-risk state of exercise addiction (Gauld et al., 2023[17]). Although the majority of the other participants showed signs of exercise addiction, none of them classified as severe exercise addiction (Gauld et al., 2023[17]). Nevertheless, these individuals are at risk of switching to problematic activity (Gauld et al., 2023[17]). Illness during pre-race periods significantly negatively impacts running performance (Gauffin et al., 2019[16]). Athletes without illness during the training phase are likelier to achieve their performance outcome than runners who suffer from pre-race illness (Gauffin et al., 2019[16]). Injured runners were shown to complete more miles per week, to be heavier, taller, and less tough-minded and forthright than the non-injured (McKelvie et al., 1985[53]). 

Motivation for endurance running may differ between sex and different age groups, however whether there are changes in motivation between different performance groups is yet not entirely clear (Nikolaidis et al., 2018[60]). Primary motivation reported amongst ultra-endurance athletes was to achieve personal goals, followed by health-related and self-esteem reasons as well as contact with nature, whereas competitive reasons were the least important motivation for taking part in ultra-marathon running (Kazimierczak et al., 2019[31]; Krokosz et al., 2018[38]; Roebuck et al., 2018[69]). Competitive marathon runners were shown to be more motivated in terms of performance level than recreational or non-athletes (Masters et al., 1993[51]; Nikolaidis et al., 2019[58]). Similar results have been previously reported in elite football players, who showed higher levels of motivation compared to recreational athletes (Nikolaidis et al., 2019[58]). Interestingly, social aspects, i.e., meeting people and socializing, motivate individuals to participate in ultra-marathon running (Krokosz et al., 2018[38]) as it helps strengthen social identity (Kazimierczak et al., 2019[31]). Energetic arousal and hedonic tone are significantly associated with ultra-marathon runners socializing motives. The more meaningful these goals were, the happier and more vigorous the athletes (Krokosz et al., 2018[38]). In amateur marathon runners, the most important motive for participation is general health orientation followed by personal goal orientation, self-esteem, psychological coping, weight concern and life-meaning (Malchrowicz-Mośko et al., 2020[46]). The lowest scores were observed in the affiliation, competition and recognition (Malchrowicz-Mośko et al., 2020[46]). However, the main motivational aspects for participating in a marathon race were not connected to race performance (see Table 3(Tab. 3)) (Malchrowicz-Mośko et al., 2020[46]). Family life and relationships influence the motivations of ultra-marathon runners (Malchrowicz-Mośko and Waśkiewicz, 2020[50]). A recent study investigating a group of ultra-marathon runners found that 3.8 % of runners had parted with their partners because they did not run or supported the passion for running (Malchrowicz-Mośko and Waśkiewicz, 2020[50]). Runners who parted with their partners showed higher scores in psychological coping, health orientation, and life meaning than their non-parting controls (see Table 4(Tab. 4)) (Malchrowicz-Mośko and Waśkiewicz, 2020[50]). The possibility of sharing leisure time with friends, family, and partners was more important to runners in a relationship and/or marriage compared to ultra-runners who parted with their partners (Malchrowicz-Mośko and Waśkiewicz, 2020[50]). 

Conclusively, endurance athletes need a certain mental strength to successfully compete in a marathon or ultra-marathon. Various aspects such as mental toughness, hardy personality, or self-efficacy also contribute to better performance. Accordingly, personality differences can also be observed between elite and recreational athletes.

## Discussion

Since the last few years, there has been a lot of interest and research in personality traits and psychological aspects of endurance runners. The human endurance capacity and challenges have contributed to the growing interest in understanding the motivational aspects of marathon, ultra-marathon, and triathlon runners concerning sex, age, and performance level, which have been studied in more detail. The purpose of this study was to review the personality traits of endurance athletes with an emphasis on recent and updated literature. 

As the main findings of this study (see Table 5(Tab. 5)), we identified a distinct and individual psychological profile of marathon runners characterized by a strong sense of vigor, self-sufficiency, intelligence, introversion and low scores in anger, fatigue, tension, and depression. This specific profile also differs from other athletes, especially joggers, ultra-marathon runners to a certain extent, cross-country skiers, and soccer players. Furthermore, endurance exercise has been identified as an important factor in the prevention of depressive symptoms, especially in breast cancer survivors. Differences between marathon runners in terms of sex were also identified, with female ultra-marathon runners showing high values in task orientation and differed in terms of motivation. In addition, differences were found between age groups: young runners were more focused on results, whereas older runners valued social interaction more. The performance level also showed differences in terms of personalities. High levels of hardy personality, mental toughness, self-efficacy, and high emotional stability can be found in endurance runners (Brace et al., 2020[4]; Nudel et al., 1989[62]; Sánchez et al., 2009[73]). 

Nevertheless, this review also has its limitations. First and foremost, the study population was very heterogeneous. Endurance runners differ drastically in terms of training background, physically but also psychologically. In addition, significantly more men continue to take part in these events, which makes generalization difficult in some cases. In addition, the question often arises as to whether personality traits are effectively a consequence of the marathon or rather a prerequisite for the sport, making comparability with other sports more difficult. Also, important to consider is the lack of quality assessment for the studies included. It is an important limitation since selection bias can impair the generalization of the findings. Considering different performance levels is also a point as different terms have been used interchangeably without a clear concept or differentiation between them, which also impairs the clinical practice (Scheer et al., 2020[74]). 

In another way, some strengths should be considered. We provided an updated overview of various aspects of marathon, ultra-marathon, and triathlon in terms of personality traits and psychological aspects. Given the high popularity of marathons among recreational and elite athletes and researchers, these findings have several practical and theoretical implications. Sport psychologists would benefit from the findings of the present study to develop psychological interventions considering runners' personality to optimize participation and performance in a marathon race. Considering the differences in sex, age, and performance listed here, it is possible and important to develop individualized training plans because these groups have different goals and needs. 

Nevertheless, many aspects remain unclear. Changes in cognition and mood states before, during, and after marathon races are not well understood and would be of great clinical importance. Additionally, large-scale studies comparing personality differences with regard to sex, age, performance, and other types of sport are rare and would corroborate these findings, highlighting the need for further original research on the personality traits of marathon runners. 

## Declaration

### Author contributions

LB, PTN and BK conceived and designed the study. LB and PTN collected data. LB, MT, CABL, VS and PTN analyzed and interpreted the data and drafted the manuscript. LB, MT, CABL, VS, PTN, KW and BK revised the manuscript and approved the final version.

### Funding

This research received no external funding.

### Conflicts of interest

The authors declare that they have no conflict of interest.

## Figures and Tables

**Table 1:**
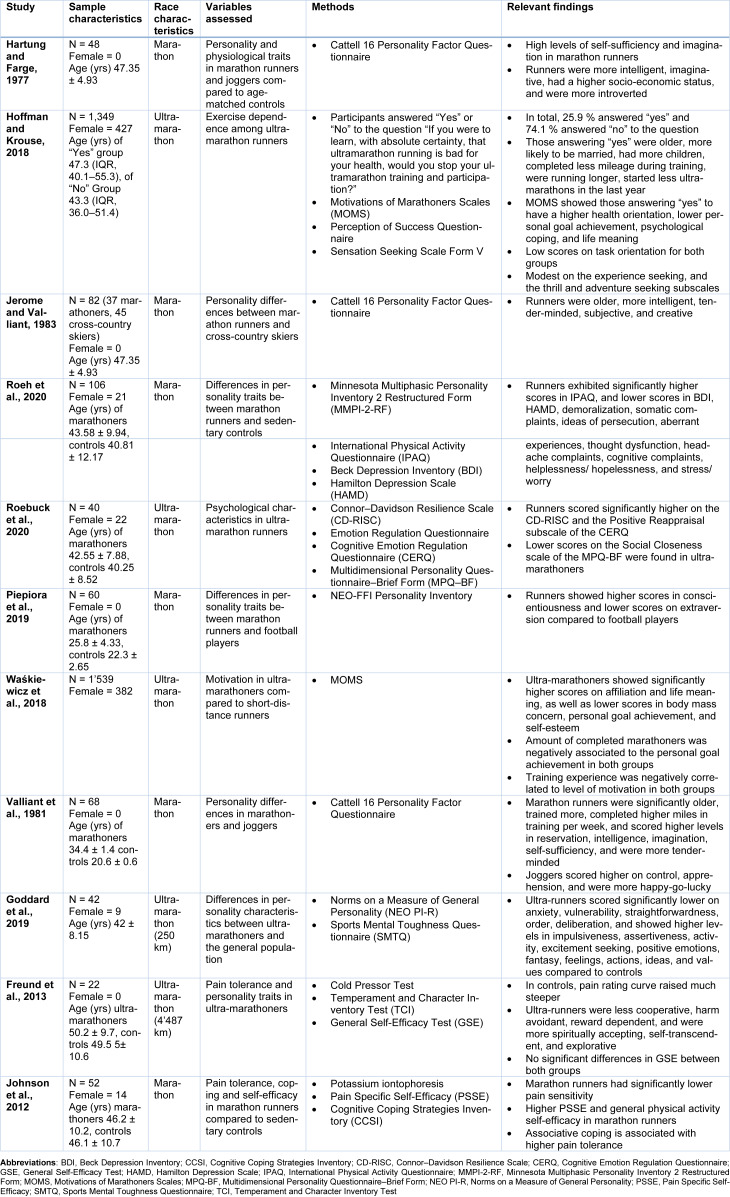
Summary of the study participants, methods, and key findings of the most important studies regarding psychological profile in endurance athletes

**Table 2:**
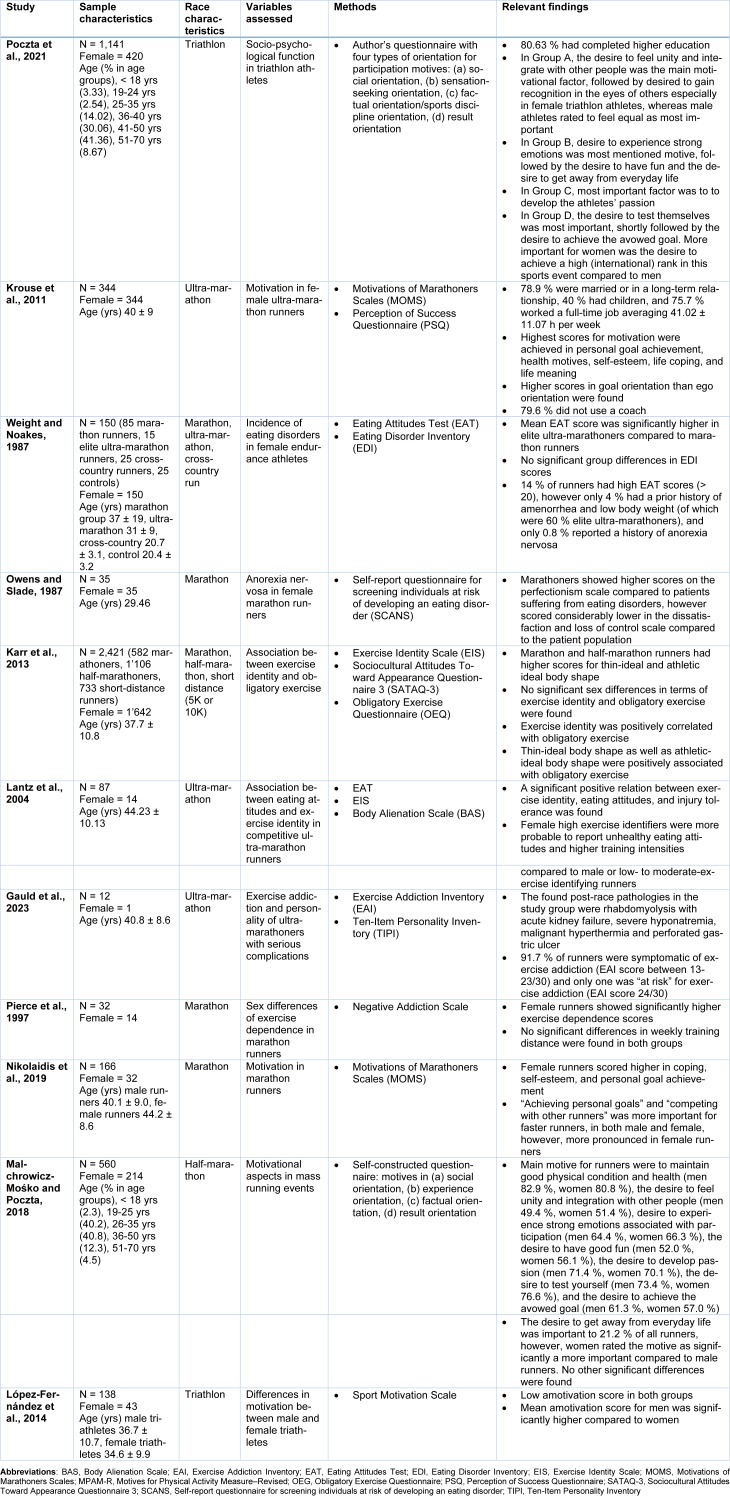
Summary of the study participants, methods, and key findings of the most important studies regarding sex in endurance athletes

**Table 3:**
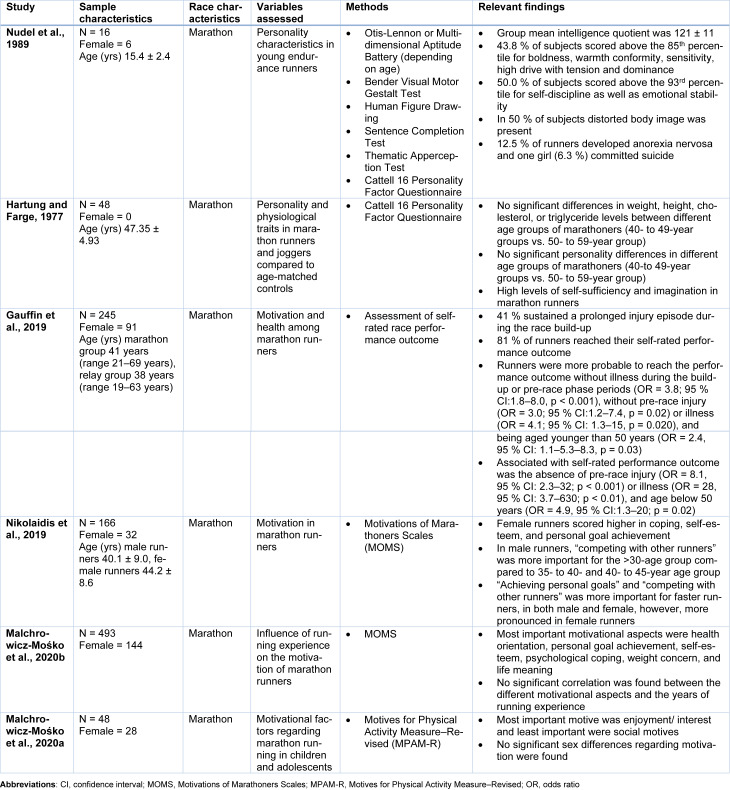
Summary of the study participants, methods, and key findings of the most important studies regarding age in endurance athletes

**Table 4:**
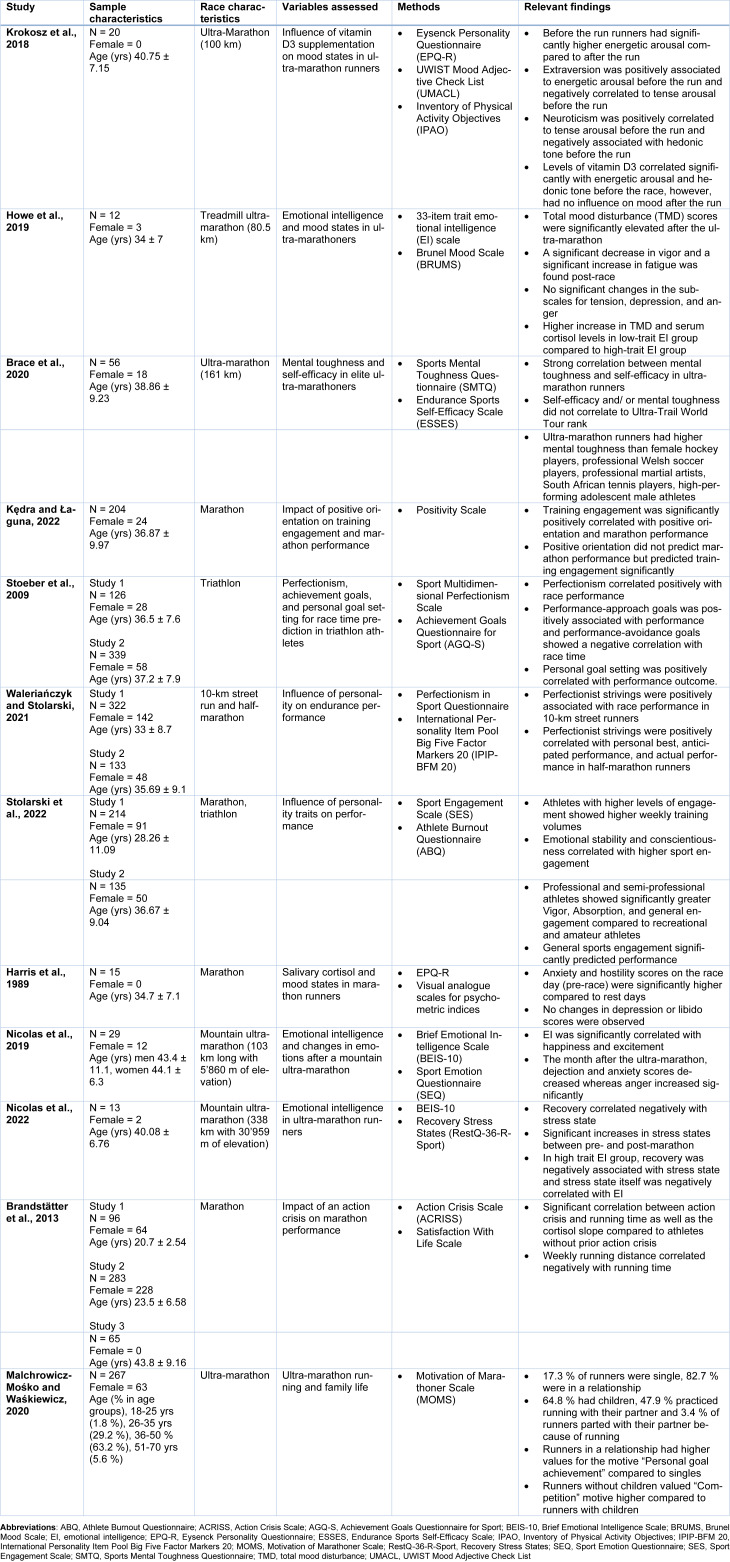
Summary of the study participants, methods, and key findings of the most important studies regarding performance level in endurance athletes

**Table 5:**
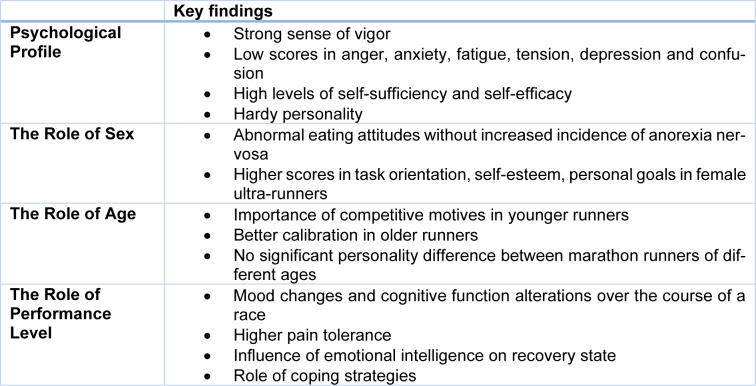
Overview of the most important aspects regarding personality of marathon runners

**Figure 1:**
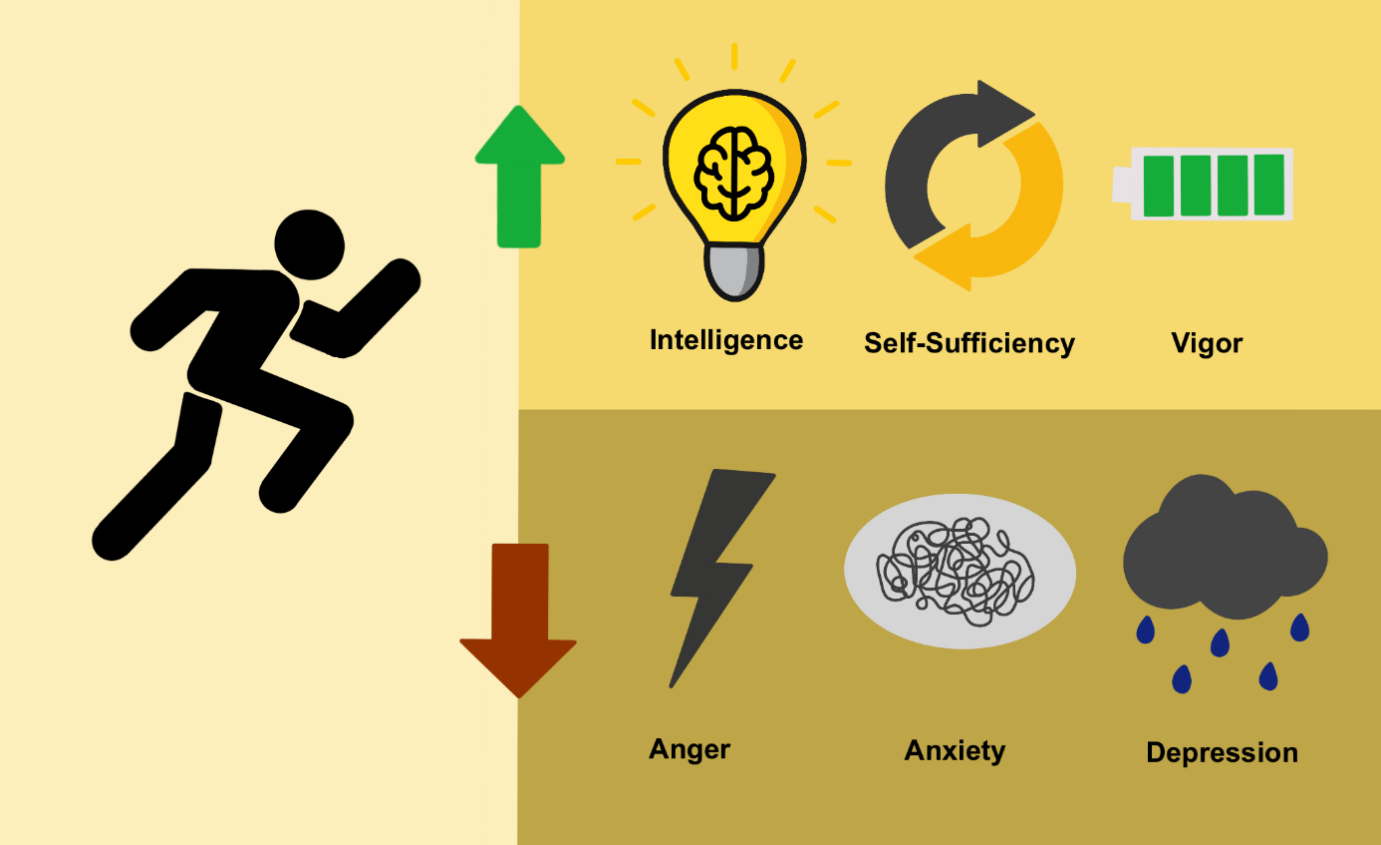
Graphical abstract
